# Network Properties in Transitions of Consciousness during Propofol-induced Sedation

**DOI:** 10.1038/s41598-017-15082-5

**Published:** 2017-12-01

**Authors:** Minji Lee, Robert D. Sanders, Seul-Ki Yeom, Dong-Ok Won, Kwang-Suk Seo, Hyun Jeong Kim, Giulio Tononi, Seong-Whan Lee

**Affiliations:** 1 Department of Brain and Cognitive Engineering, Korea University, Seoul, 02841 Korea; 2 Department of Anesthesiology, University of Wisconsin, Madison, Wisconsin 53792 USA; 3 Department of Dental Anesthesiology, Seoul National University Dental Hospital, Seoul, 03080 Korea; 4 Department of Psychiatry, University of Wisconsin, Madison, Wisconsin 53719 USA

## Abstract

Reliable electroencephalography (EEG) signatures of transitions between consciousness and unconsciousness under anaesthesia have not yet been identified. Herein we examined network changes using graph theoretical analysis of high-density EEG during patient-titrated propofol-induced sedation. Responsiveness was used as a surrogate for consciousness. We divided the data into five states: baseline, transition into unresponsiveness, unresponsiveness, transition into responsiveness, and recovery. Power spectral analysis showed that delta power increased from responsiveness to unresponsiveness. In unresponsiveness, delta waves propagated from frontal to parietal regions as a traveling wave. Local increases in delta connectivity were evident in parietal but not frontal regions. Graph theory analysis showed that increased local efficiency could differentiate the levels of responsiveness. Interestingly, during transitions of responsive states, increased beta connectivity was noted relative to consciousness and unconsciousness, again with increased local efficiency. Abrupt network changes are evident in the transitions in responsiveness, with increased beta band power/connectivity marking transitions between responsive states, while the delta power/connectivity changes were consistent with the fading of consciousness using its surrogate responsiveness. These results provide novel insights into the neural correlates of these behavioural transitions and EEG signatures for monitoring the levels of consciousness under sedation.

## Introduction

Understanding the mechanisms of the transitions between consciousness and unconsciousness is a challenge for modern neuroscience particularly as behavioural report is the gold standard (some may argue only) measure of consciousness. In this regard most studies of anaesthetic-induced changes in consciousness, infer this state from changes in behavioural responsiveness^1,2^. Reversible changes in consciousness, based on responsiveness, appear to correlate with changes in slow wave activity, measured by recording the power between 0.5 and 4 Hz (delta activity)^3,4^. In other words, it is well known the delta activity is a typical feature as a signature of unconsciousness^5^. Hypothetically, increased delta activity leads to altered cortical information processing and connectivity^6^, perhaps reflecting a breakdown of integrated functional networks within the brain^7^ and a disruption of connectivity in the fronto-parietal network^8,9^. The alpha and beta power increase with the infusion of propofol, similar to the delta activity^10,11^ with reversed changes of brain dynamics occur during recovery in the delta, alpha, and beta bands^12–14^. However, the mechanisms of brain network changes during these state transitions is unclear^15,16^. Specifically, the changes of brain activity and connectivity at the critical points during transitions of consciousness are not known.

Graph-theoretical network analysis used in the representations of networks as a method to represent gradual or abrupt changes in brain networks during transition into unconsciousness^9,17^. Indeed sensitivity to abrupt changes in network dynamics will likely be critical to the disintegration of consciousness^1^ or at least, the state transition associated with the unresponsive state^18^. Graph theory provides a way to quantify these changes across static levels of consciousness.

During the transition into unconsciousness, the electroencephalography (EEG) delta and alpha band topology and connectivity changes^7,14^. The alpha connectivity is reduced during ketamine-induced unresponsiveness compared to that during wakefulness^19^. The functional network in the alpha band is also decreased during sevoflurane anesthesia including transition into conscious or unconscious states^20^. During propofol-induced sedation, the alpha connectivity is compromised and is different from that during wakefulness^21^. The network properties in the alpha band are reverted during recovery^9,22^. However, the network dynamics during transitions into unconsciousness are poorly understood.

Recent studies have indicated major interest in the main brain regions associated with the transitions of consciousness. It is reported that delta activity migrates from the parietal to the frontal region during unconsciousness^12^. The alpha and beta activities in the frontal region also increase during anaesthetic-induced EEG changes. Due to these changes called ‘frontal predominance’, the frontal region was thought to be important for altering consciousness^12^. The central roles are replaced from the parietal hub to frontal hub in the alpha network during unconsciousness^22^ though these may not be causally linked to changes in consciousness^23^. The possibility of this causal discrepancy is explained that the delta activity propagates from frontal to parietal regions during sleep as a traveling wave^24^. Indeed in some cases, consciousness is maintained after widespread frontal lesions^25^ and patients may be conscious and able to communicate to the outside world under anaesthesia with profound frontal delta activity^26^. Hence the relevance of changes in the frontal and parietal regions during the depths of consciousness remains to be confirmed.

This present study investigated the changes in topological properties of functional brain networks during propofol-induced unconsciousness induced by a novel research method, patient-controlled sedation. Patient-controlled sedation is an effective way to produce rapid and reversible changes in the conscious state, assessed through behavioural responsiveness, with the subject controlling the drug administration^27^. Hence, it is possible to study the transitions in the conscious state through repeated transitions within a single subject. We applied high-density EEG during intravenous anaesthesia with propofol. Propofol is broadly employed in medical settings to induce a state of reduced behavioural responsiveness as in non-rapid eye movement sleep^28^. We hypothesized that there would be variation in the functional reorganization of neuronal networks at differing depths of propofol-induced sedation. Furthermore, we expected differences in features of the frontal and parietal regions during the levels of consciousness. These findings may be key signatures of network properties during transitions between consciousness and unconsciousness, revealed by changes in responsiveness.

## Results

### The pharmacological and behavioural changes during propofol-induced sedation

**Figure 1:**
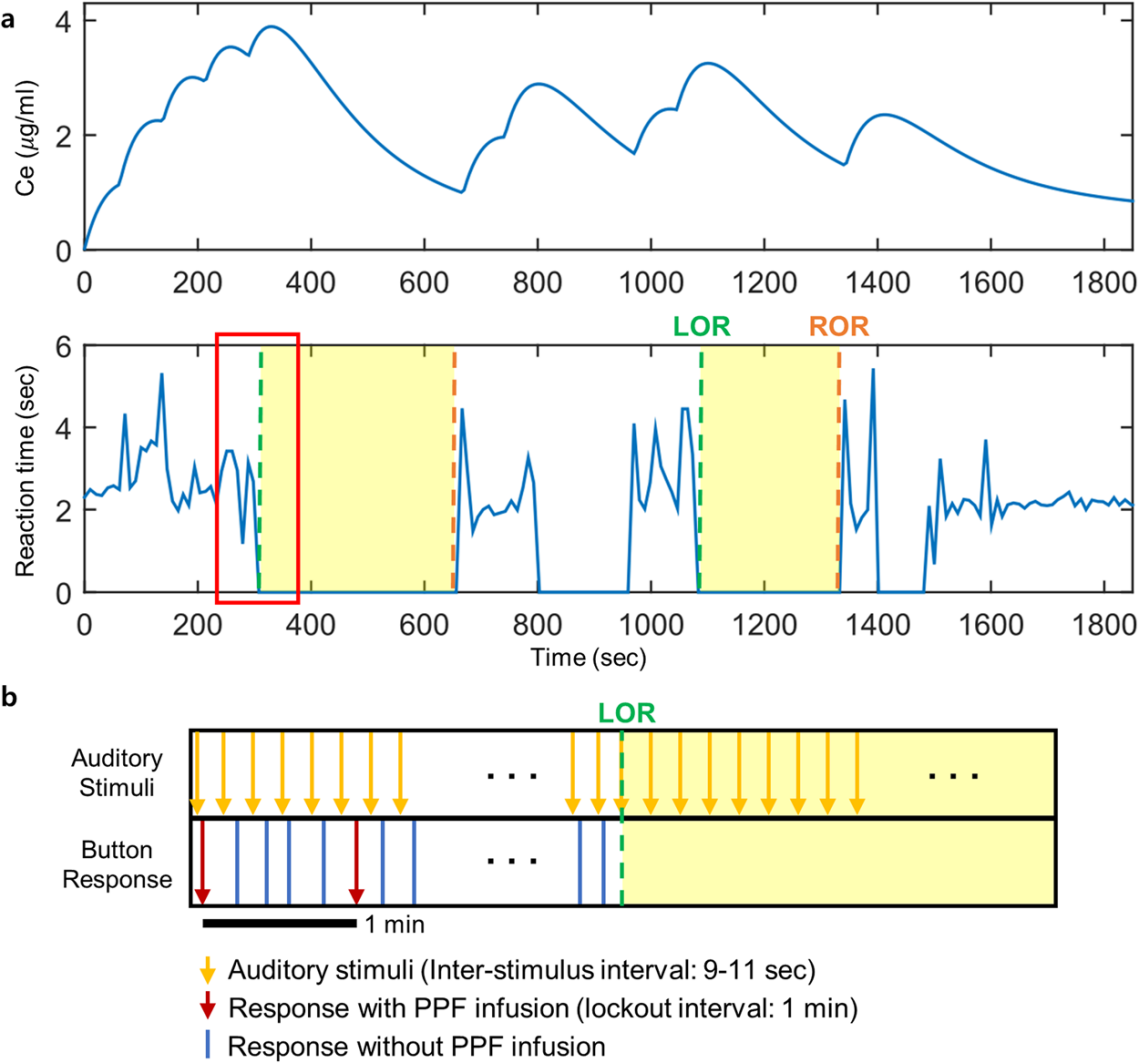
Pharmacological and behavioural profile based on patient-controlled sedation: (**a**) Infusion of PPF is initiated when the subject presses the administration button in response to auditory stimuli. On achieving individual titer, the subject is deeply sedated and does not respond. As PPF is not infused further, the concentration of PPF decreases. After ROR, the subject presses the administration button again. (**b**) A detailed examination of the red box of (**a**) reveals that the inter-stimulus interval is 9–11 sec. However, PPF is not injected each time the subjects press the button due to a lock-out time of 1 min to prevent the overdose of PPF. In other words, the PPF can be injected again after 1 min of response with PPF infusion. The data are derived from a representative subject. The green vertical line is the point of LOR. The orange vertical line indicates the point of ROR. The yellow sections represent unconscious states. In this study, the unconscious state was regarded as comprising of at least 3 min extending from LOR to ROR. Ce, effect site concentrations; LOR, loss of responsiveness; ROR, recovery of responsiveness; PPF, propofol.

We investigated the change of functional dynamics during transitions of responsiveness. Ten healthy subjects completed an auditory task with a mean inter-stimulus interval of 10 seconds composed of interleaved verbal and click stimuli. Pharmacological and behavioural changes during propofol-induced sedation are shown in Fig. 1. We defined responsiveness as behavioral response to auditory stimuli. Loss of responsiveness (LOR) was regarded as the first non-response to the auditory stimulus during induction, and recovery of responsiveness (ROR) was defined as the first response to the auditory stimulus after LOR. We defined that the unresponsive state should comprise of at least 3 min extending from LOR to ROR. We divided the five states of responsiveness based on behavioural changes as follows: (1) Baseline: the 100% responsive state before propofol administration, (2) Transition to unresponsiveness (Trans_UN_): the transition state to LOR during induction, (3) Unresponsiveness: the non-responsive state at the midpoint between LOR and ROR, (4) Transition to responsiveness (Trans_RES_): the transition state to ROR during emergence, (5) Recovery: the 100% responsive state after propofol administration. The spectra power for six frequency bands (delta, theta, alpha, sigma, beta, and gamma) with the various levels of responsiveness was calculated in the frontal and parietal regions. The frontal region was defined as 13 channels (FP1–2, AF3–6, F1–4, F7–8, and Fz), whereas the parietal region was regarded as 14 channels (P1–8, Pz, PO3–4, PO7–8, and POz) according to the international 10–10 electrode system. To determine the changes of functional networks, the connectivity estimator between two signals was also measured using frequency-wise, region-wise weighted Phase Lag Index (wPLI). The wPLI, a functional connectivity measure, was calculated from a phase difference weighting normalization as the magnitude of the imaginary part of the cross-spectrum. Global efficiency (a measure of global integration), local efficiency (a measure of local segregation), and small-worldness (a combined measure of the local and global efficiencies) based on wPLI were computed (see Methods for more details).

### The spectral dynamics with the states of responsiveness

**Figure 2:**
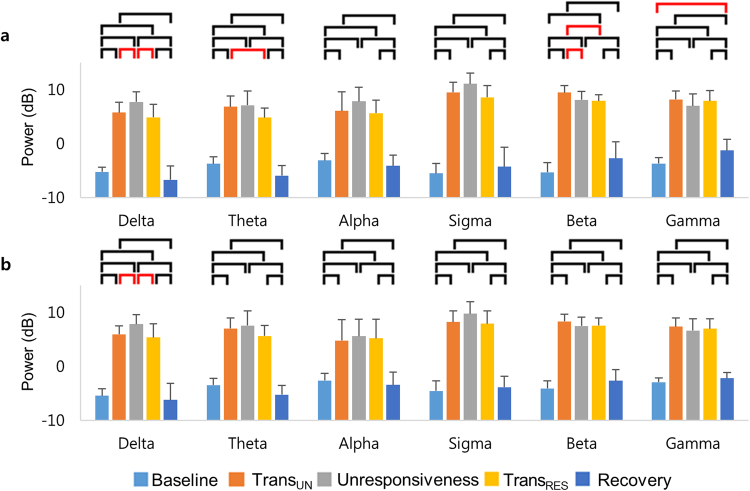
The changes of the averaged power spectra with the levels of responsiveness: The power spectra in (**a**) frontal and (**b**) parietal regions average across subjects and for all frequencies. The frontal region refers to 13 channels (FP1–2, AF3–6, F1–4, F7–8, and Fz), whereas the parietal region refers to 14 channels (P1–8, Pz, PO3–4, PO7–8, and POz). Error bars indicate standard deviations across all subjects. The black horizontal bars indicate the significant differences between responsive and unresponsive states, and the red horizontal bars represent other significant differences for paired t-test with Holm-Bonferroni correction (*p* < 0.05). Trans_UN_ = transition into unresponsiveness, Trans_RES_ = transition into responsiveness.

Figure 2 represents the changes of the averaged spectral dynamics over the frontal and parietal regions. As unresponsiveness changed from responsiveness, the spectra power over the frontal region increased in the delta (*F*_(4, 45)_ = 111.64, *p* < 0.001), theta (*F*_(4, 45)_ = 98.76, *p* < 0.001), alpha (*F*_(4, 45)_ = 51.2, *p* < 0.001), sigma (*F*_(4, 45)_ = 112.5, *p* < 0.001), beta (*F*_(4, 45)_ = 134.62, *p* < 0.001), and gamma (*F*_(4, 45)_ = 96.43, *p* < 0.001) bands (Fig. 2a). Specifically, the power during the unresponsive states (Trans_UN_, unresponsiveness, and Trans_RES_) was significantly higher than that during the conscious states (baseline and recovery) across all frequencies. The delta power in unresponsiveness was significantly higher than those in the transitions of responsiveness (Trans_UN_ and Trans_RES_). The power spectra in Trans_UN_ was significantly higher than that in unresponsiveness for the beta band. The difference of power between baseline and recovery for the gamma band was significantly explored. Lastly, we observed a significant difference between Trans_UN_ and Trans_RES_ for the theta and beta bands.

Similar to frontal regions, changing from responsiveness to unresponsiveness, the power spectra over the parietal region was higher in the delta (*F*_(4, 45)_ = 100.71, *p* < 0.001), theta (*F*_(4, 45)_ = 97.87, *p* < 0.001), alpha (*F*_(4, 45)_ = 22.98, *p* < 0.001), sigma (*F*_(4, 45)_ = 113.51, *p* < 0.001), beta (*F*_(4, 45)_ = 149.36, *p* < 0.001), and gamma (*F*_(4, 45)_ = 109.73, *p* < 0.001; Fig. 2b) bands. The delta power in unresponsiveness was significantly higher than those in the transitions of responsiveness (Trans_UN_ and Trans_RES_) (Supplementary Table S1).

### The changes of wPLI during the levels of responsiveness

**Figure 3:**
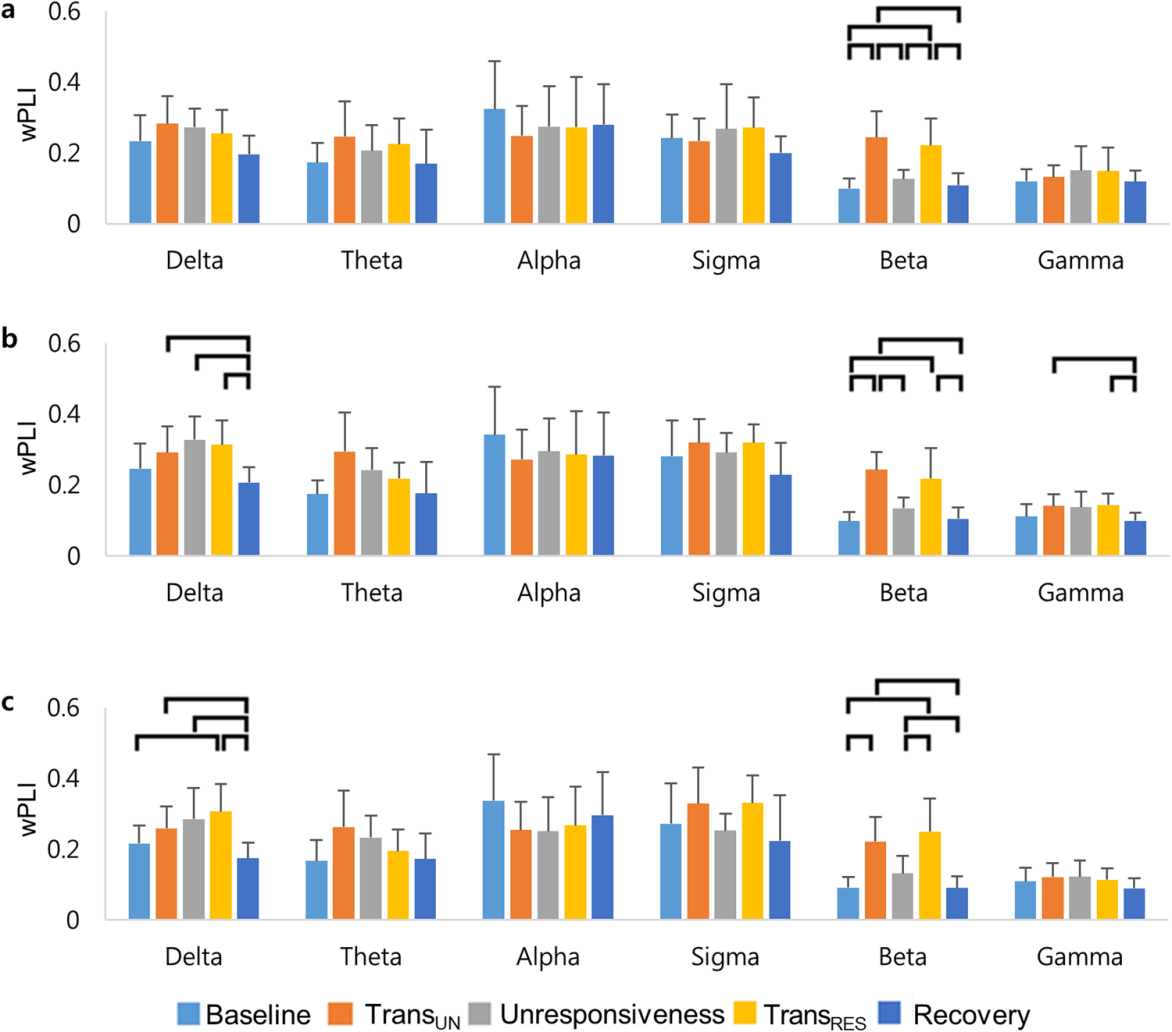
The changes of the averaged wPLI during propofol-induced sedation: (**a**) frontal wPLI, (**b**) fronto-parietal wPLI, and (**c**) parietal wPLI of all frequencies. The frontal region refers to 13 channels (FP1–2, AF3–6, F1–4, F7–8, and Fz), whereas the parietal region refers to 14 channels (P1–8, Pz, PO3–4, PO7–8, and POz). Error bars indicate standard deviations across all subjects. The horizontal bars show the significant differences for paired t-test with Holm-Bonferroni correction (*p* < 0.05). Trans_UN_ = transition into unresponsiveness, Trans_RES_ = transition into responsiveness.

A change of wPLI was found with the states of responsiveness. The averaged wPLI for the delta and beta bands, in particular, showed significant changes in the different forms of the levels of responsiveness (Fig. 3). In each comparison, a one-way analysis of variance (ANOVA) revealed the increased wPLI in the frontal region for the beta band during transitions in and out of unresponsiveness (*F*_(4, 45)_ = 16.74, *p* < 0.001). The fronto-parietal connectivity measured by wPLI demonstrated a significant change for the delta (*F*_(4, 45)_ = 5.95, *p* < 0.001), beta (*F*_(4, 45)_ = 17.9, *p* < 0.001), and gamma (*F*_(4, 45)_ = 3.45, *p* = 0.015) bands. The delta and gamma wPLI increased during unresponsiveness, whereas beta wPLI was higher in transition points than other three points in fronto-parietal connectivity. There were also significant differences in the parietal region for the delta (*F*_(4, 45)_ = 6.4, *p* < 0.001) and beta (*F*_(4, 45)_ = 15.4, *p* < 0.001) bands during the levels of responsiveness.

A pivotal finding is that the delta wPLI during unresponsiveness increased over the parietal region but not the frontal region during unresponsiveness. A post hoc paired t-test indicated that the wPLI during recovery was significantly lower than those during Trans_UN_, unresponsiveness, and Trans_RES_ in the parietal region and fronto-parietal interaction for the delta band. The fronto-parietal and parietal delta connectivity were increased from responsiveness to unresponsiveness. In other words, propofol caused stronger fronto-parietal and parietal connectivity in the delta band. To explore the clear changes of delta wPLI between responsiveness and unresponsiveness, we compared the baseline, unresponsive states (the average value of Trans_UN_, unresponsiveness, and Trans_RES_), and recovery. For convenience, we defined UCS as including unresponsiveness, and transitions into unresponsiveness or responsiveness. The delta wPLI was significantly changed in frontal (*F*_(2, 27)_ = 4.02, *p* = 0.030), fronto-parietal (*F*_(2, 27)_ = 8.54, *p* = 0.001), and parietal regions (*F*_(2, 27)_ = 15.79, *p* < 0.001), respectively (Supplementary Table S2). Specifically, the delta wPLI in UCS was higher compared to recovery regardless of the area of the brain, whereas the delta wPLI in UCS was significantly higher than baseline only in the parietal region (Supplementary Fig. S1). The wPLI during the transitions into and out of responsiveness was significantly higher than that in the other states (baseline, unresponsiveness, and recovery) in the frontal and parietal regions and fronto-parietal interaction for the beta band. In other words, the beta wPLI during the transitions of responsiveness was the highest over the frontal, parietal, and fronto-parietal connectivity. During propofol-induced transitions between responsiveness and unresponsiveness, the beta activity was increased. For the gamma band, the wPLI was also significantly higher during Trans_RES_ than during Trans_UN_ and recovery (Supplementary Table S3). Supplementary Figure S2 illustrates the change of wPLI at the individual levels in the frontal and parietal regions and the fronto-parietal interaction (see supplementary discussion about individual data).

We also observed a correlation between the power spectra and connectivity (Table 1). The linear correlation in the frontal and parietal regions was significantly explored for the delta, theta, and beta bands. Based on these results, we focused on changes in the delta and beta bands in further analysis.

**Table 1:** The statistical value in the linear correlation between power and wPLI. The asterisk represents the significance with Holm-Bonferroni correction (p < 0.05).

Region	Frequency	Delta	Theta	Alpha	Sigma	Beta	Gamma
	Value						
Frontal region	*r*	0.405	0.402	−0.042	0.260	0.619	0.268
	*p*	0.003*	0.003*	0.774	0.068	<0.001*	0.060
Parietal region	*r*	0.513	0.442	−0.192	0.373	0.626	0.369
	*p*	<0.001*	0.001*	0.182	0.007*	<0.001*	0.008*

**Figure 4:**
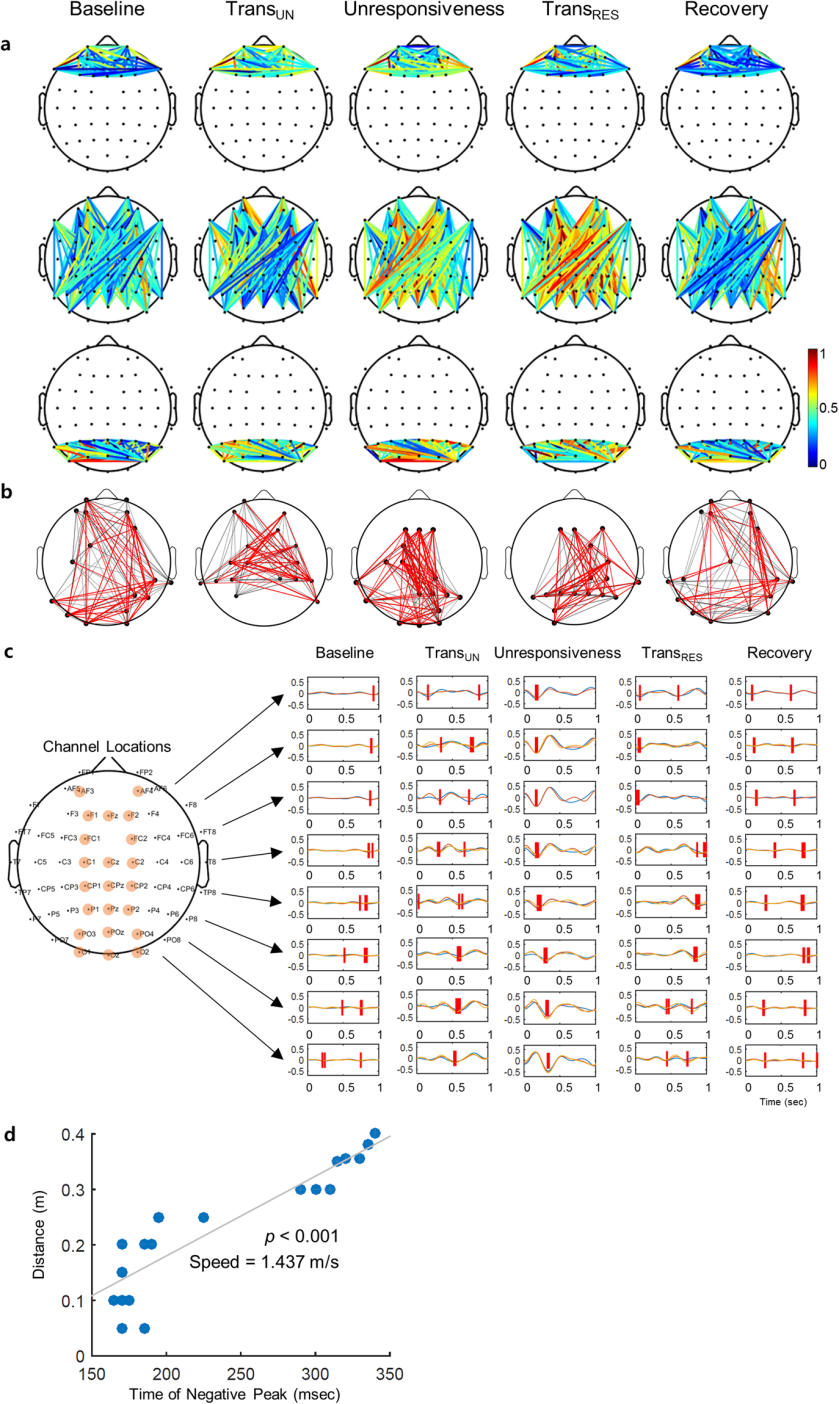
The changes of delta connectivity across five states: (**a**) The frontal (up), fronto-parietal (middle), and parietal wPLI (down) are plotted. The connection strength is wPLI. The colour map represents the value of wPLI. The frontal region refers to 13 channels (FP1–2, AF3–6, F1–4, F7–8, and Fz), whereas the parietal region refers to 14 channels (P1–8, Pz, PO3–4, PO7–8, and POz). (**b**) For visual clarity, only the strongest 30% of the connections (wPLI) are depicted. The node of the network refers to the degree, whereas the edge of the network represents the weights of wPLI. The red colour of the edges indicates strong connectivity, whereas the gray colour indicates weak connectivity. (**c**) The delta wave on the midline channels (orange circles) is plotted. The red lines represent the negative peak points of delta waves. (**d**) Correlation between the distance on the scalp in the anterior-posterior axis and the time for negative peak and the speed of wave propagation are indicated during unresponsiveness. Trans_UN_ = transition into unresponsiveness, Trans_RES_ = transition into responsiveness.

The changes of functional network in the delta connectivity using wPLI between two signals during the levels of responsiveness were showed. All connections were stronger during unresponsiveness in the parietal region and fronto-parietal interaction for the delta band (Fig. 4a). The changes of the brain networks, composed of nodes (degree) and edges (the weight of wPLI), was investigated during propofol-induced sedation. At baseline, there were weak fronto-parietal connections and a cluster in the parietal region for the delta band. The fronto-parietal interaction was more intense during the transition into unresponsiveness than at the baseline. The delta connectivity was the strongest during propofol-induced unresponsiveness. The functional networks at recovery returned to the typical pattern of the baseline level (Fig. 4b). Specifically, we measured the traveling delta wave to explore the role of a traveling wave related in consciousness. Figure 4c showed the delta wave on the midline channels during the levels of responsiveness. As a result, there was the clear traveling wave from frontal to the parietal direction during unresponsiveness. The negative peak, which is the critical measure for indicating the brain dynamics of the delta wave, moved posteriorly in only unresponsiveness. The significant correlation between the anterior-posterior axis and time for negative peak was investigated in the unresponsiveness (*r* = 0.908, *p* < 0.001). The velocity of the wave was 1.437 m/s (Fig. 4d) (see more the detail in supplementary methods).

**Figure 5:**
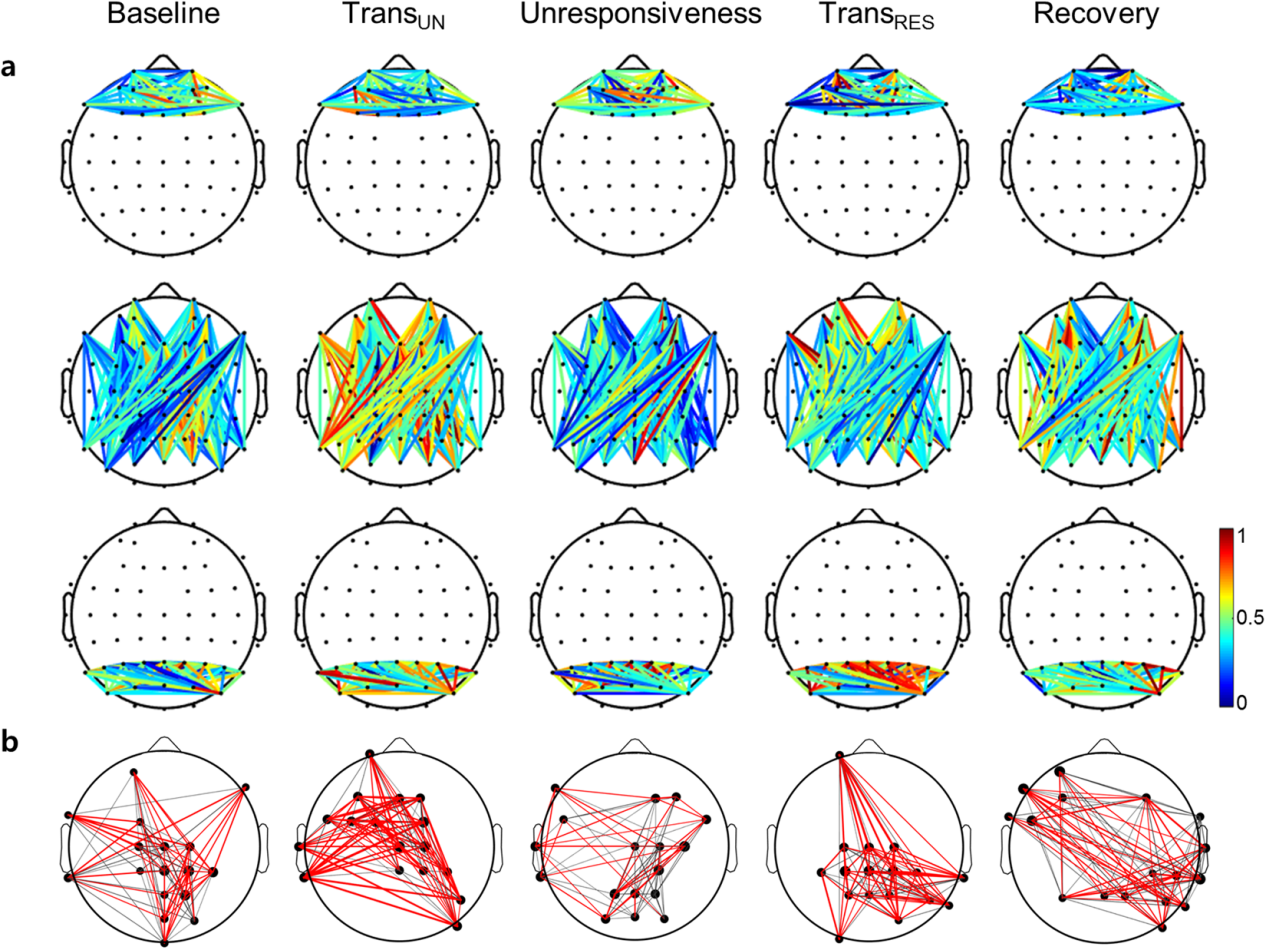
The changes of beta connectivity across five states: (**a**) The frontal (up), fronto-parietal (middle), and parietal wPLI (down) are plotted. The connection strength is wPLI. The colour map represents the value of wPLI. The frontal region refers to 13 channels (FP1–2, AF3–6, F1–4, F7–8, and Fz), whereas the parietal region refers to 14 channels (P1–8, Pz, PO3–4, PO7–8, and POz). (**b**) For visual clarity, only the strongest 30% of the connections (wPLI) are depicted. The node of the network refers to the degree, whereas the edge of the network represents the weights of wPLI. The red colour of the edges indicates strong connectivity, whereas the gray colour indicates weak connectivity. Trans_UN_ = transition into unresponsiveness, Trans_RES_ = transition into responsiveness.

On the contrary to delta connectivity, the links during the transition points of responsiveness were stronger for the beta band in all frontal and parietal regions including fronto-parietal interaction (Fig. 5a). Figure 5b depicts the changes of functional networks for the beta band with the levels of responsiveness. In the beta band, the functional connectivity was strengthened at the moment of transition into unresponsiveness and weakened again with unresponsiveness. At the moment of recovering responsiveness, the beta connectivity was stronger and was reduced to the baseline level during the recovery. There was a characteristic increase in beta connectivity at the transition points between responsiveness and unresponsiveness. Of course, the beta connectivity in unresponsiveness may look somewhat weaker than in baseline or recovery, but statistically, there was no difference between them.

### The changes of network properties during propofol-induced sedation

**Figure 6:**
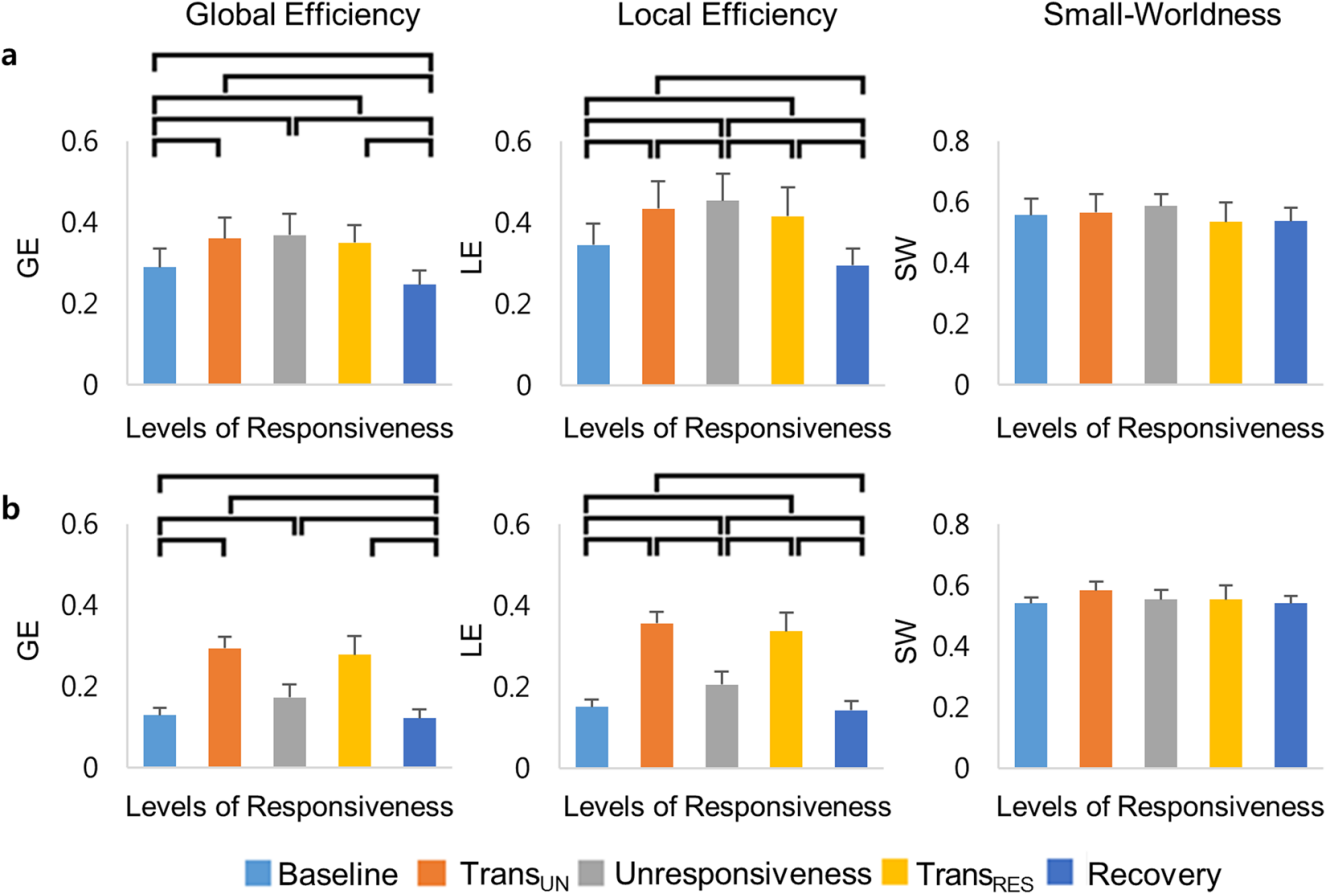
The changes of network properties with the levels of consciousness: The global efficiency, local efficiency, and small-worldness for (**a**) delta band and (**b**) beta band plotted across five states (Baseline – Trans_UN_ – unresponsiveness – Trans_RES_ – Recovery). Error bars indicate standard deviations across all subjects. The horizontal bars show significant differences using paired t-test with Holm-Bonferroni correction (*p* < 0.05). GE = global efficiency, LE = local efficiency, SW = Small-Worldness, Trans_UN_ = transition into unresponsiveness, Trans_RES_ = transition into responsiveness.

The changes of network properties with the levels of responsiveness were found for the delta and beta bands (Fig. 6). We observed increased global and local efficiencies during unresponsiveness for the delta band (Global efficiency: *F*_(4, 45)_ = 12.92, *p* < 0.001, local efficiency: *F*_(4, 45)_ = 11.95, *p* < 0.001). These changes reverted to the baseline level during recovery. Significant changes of global and local efficiencies were investigated for the beta band (Global efficiency: *F*_(4, 45)_ = 69.28, *p* < 0.001, local efficiency: *F*_(4, 45)_ = 52.24, *p* < 0.001). The global and local efficiencies for the beta band were the highest during propofol-induced transitions in responsiveness (Supplementary Table S4). Altogether, small-worldness, which is the combination of global and local efficiencies, was not significantly changed for the delta and beta bands.

**Figure 7:**
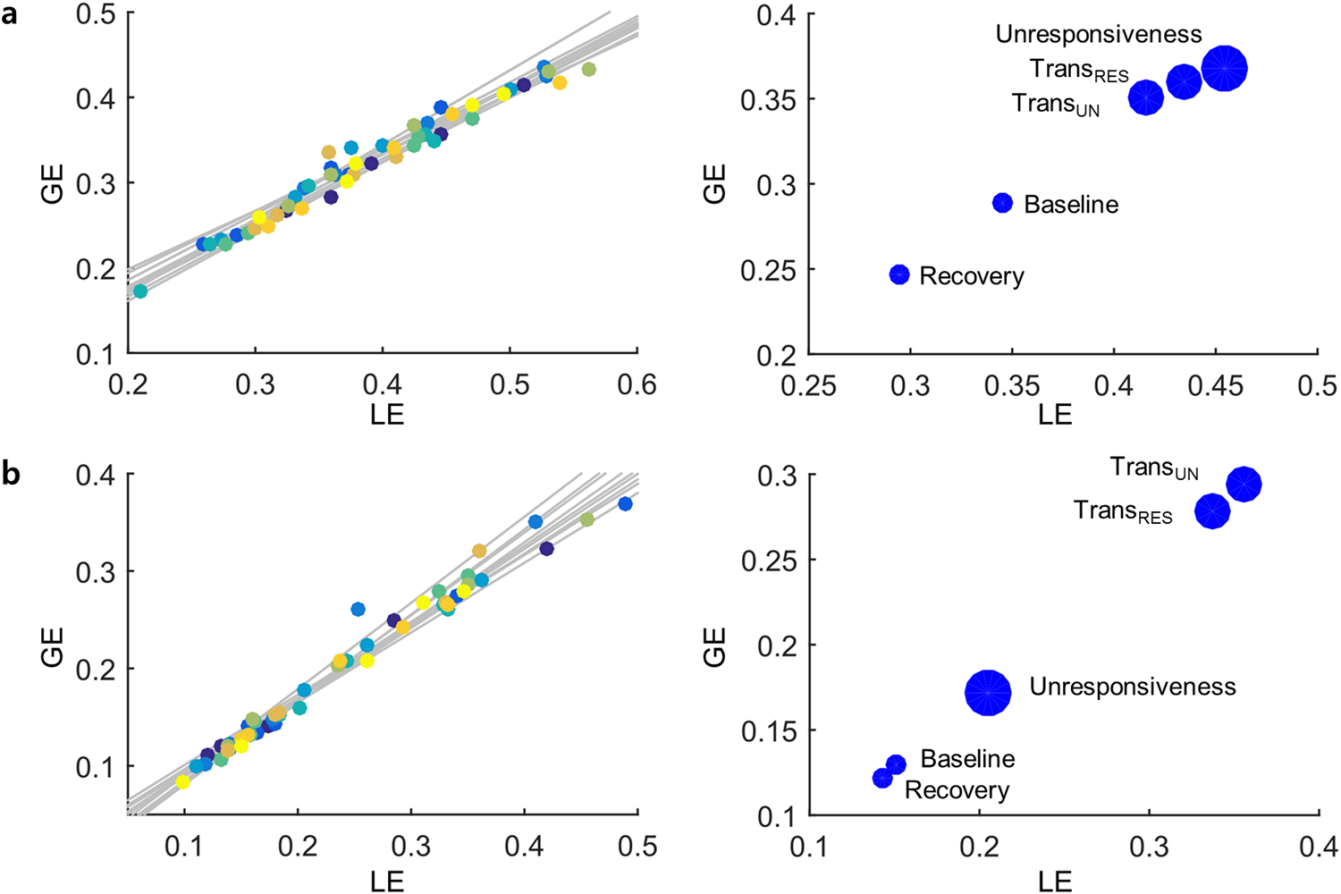
The relationship between global and local efficiencies: There is an individual correlationship between the global and local efficiencies (left) and the scatter plot between the mean global efficiency and the mean local efficiency (right) for (**a**) delta band and (**b**) beta band across the levels of responsiveness. The largest circle represents unresponsiveness, and the medium sized, the transition into and out of responsiveness. The smallest circle also represents baseline and recovery. GE = global efficiency, LE = local efficiency, Trans_UN_ = transition into unresponsiveness, Trans_RES_ = transition into responsiveness.

To further investigate this result, Fig. 7 was used to illustrate the contrast between the global and local efficiencies with states of responsiveness. It was notable that there was a positive correlation between the global and local efficiencies for all the subjects with respect to the delta band (*r* = 0.983, *p* < 0.001). There was the clear relationship between the averaged global efficiency and the averaged local efficiency for the delta band. The efficiency of the network at baseline and recovery was lower than the other states. These changes in functional efficiency could be divided into unconscious and conscious states for the delta band. A positive correlation was also observed for the beta band with the levels of responsiveness (*r* = 0.988, *p* < 0.001). The precise relationship between the averaged global efficiency and the averaged local efficiency was observed for the beta band. This characteristic could confirm the timing of the conscious transition.

## Discussion

In the present study, we explored the change in brain functional connectivity and applied graph theoretical analysis of network properties using high-density EEG. First, we found increased power in the delta band with progressive and reversible changes in consciousness; notably delta power could discriminate the transitions into and from unconsciousness. Second, increased parietal and fronto-parietal delta connectivity occurred during propofol-induced sedation. Third, the topological properties (global and local efficiencies) in the delta band were significantly increased during transition into unconsciousness. Fourth, frontal, parietal and fronto-parietal beta band wPLI were increased during the moments of transition into and out of consciousness. Positive correlations between global and local efficiencies for the delta and beta bands were strongly observed.

The delta power accorded with the levels of consciousness induced by propofol^7,15,29^. We observed the increased activity and connectivity in the delta band during unconsciousness. Bistability could be an underlying reason for how delta activity contributes to unconsciousness. The emergence of delta activity likely represents an intrinsic bistability of cortical networks, which is the transition between depolarized (up) states of and hyperpolarized (down) states. The delta waves is a major characteristic of the cortical bistabiity of brain networks during unconsciousness^30^. Under the influence of propofol, neurons continue to be silent with a prominence of delta activity^31^. Due to the leakage of depolarization-dependent potassium currents, hyperpolarized states briefly occur in thalamocortical and cortical neurons. It has been proposed that this bistability is a primary mechanism underlying both, the emergence of delta activity and the changes in information processing during unconsciousness^3,29^, likely through breaking down neuronal networks^32^. Therefore, increased delta connectivity during unconsciousness is a vital finding in terms of reflecting coordinated bistability that suppresses sufficient integrated information for consciousness.

A critical finding is that wPLI for the delta band increased during unconsciousness in the parietal region but not in the frontal region. This result is consistent with some recent evidence indicating that the frontal region is not essential for consciousness, rather a ‘posterior hot zone’ focused on the parietal regions is critical in this regard^23^. Although several studies have focused on changes in the frontal region during unconsciousness^7,31^, recent interest has focussed on more posterior regions, which are thought to play a direct role as a hub structure during transitions of consciousness^22^. The parietal region is a complex of the lateral temporo-parieto-occipital junction and a mesial cortical core. This complex appears likely to be a final general target for anaesthetic-induced unconsciousness^33^. Our recent data also suggest that profound delta power in the frontal region does not ensure unconsciousness after intubation for clinical anaesthesia; data on parietal regions from clinical studies is lacking^25^. Our data suggest that the delta waves also propagate as a traveling wave in the anteroposterior direction only during unresponsiveness. This is in line with exploring that slow oscillations periodically sweep the brain cortex and propagate in the anteroposterior direction during sleep^24^. Spontaneous delta waves in both sleep and propofol-induced anesthesia propagate along the midline highway in a posterior direction; because propofol delta waves share the components with sleep delta waves such as the anterior cingulate and posterior cingulate^28^. This traveling wave phenomenon originated from frontal regions occurs^24^ and were associated with unconsciousness when reaching posterior regions. It appears that the propagation of traveling delta waves in the anteroposterior direction is a major feature during unconsciousness and specifically that this backward propagation of bistability may lead to unconsciousness when the traveling wave reaches parietal regions.

Several studies have investigated the fronto-parietal connectivity for a central role in levels of consciousness^19,22^, however some of these changes may be epiphenomenal, and not bear a causal relationship to consciousness^34^. The functional magnetic resonance imaging studies also showed that the changes in connectivity of the parietal region are associated with the transition to unconsciousness^35,36^. In summary we conclude that parietal cortical dynamics are associated with changes in consciousness.

Interestingly, several studies have reported that beta connectivity is not altered during unconsciousness^20^. This compares favourably with our study where we observed no significant difference in beta connectivity between consciousness and unconsciousness. However, beta connectivity significantly increased during the transitions into and from unconsciousness. Our paradigm is appropriate for identifying the critical point of transitions between consciousness and unconsciousness^7^ as there are multiple state transitions. A brief peak of beta dynamics also appears during transitions into unconsciousness and the delta dynamics during unconsciousness increase in primates^37^. Regarding connectivity changes, consciousness decidedly disappears after hypersynchrony, as in the instance of generalized seizures. This hypersynchrony produces the inefficiently integrated information due to the excessively abundant brain connectivity, and the distinct specificity of each element is lost. This phenomenon continues to result in unconsciousness^38–40^. We observed hypersynchrony in the beta band during propofol-induced transitions between consciousness and unconsciousness. In particular the global and local efficiencies in the beta band increase during the critical points only when consciousness vanishes and appears. The high correlation between global and local efficiencies also clearly distinguishes the critical point of transitions between consciousness and unconsciousness. This biphasic effect, whereby beta connectivity increase initially and then decrease as soon as they reach high concentrations, seems to reflect the relationship between hypersynchrony and transitions into consciousness^41^. Our working hypothesis is that these dynamics herald the breakdown in the integration of information across a widespread region^42,43^. This may involve a critical delay in feedback processing in corticothalamic networks^44^ associated with increased beta power, leading to a “network reset” where a new state configuration is obtained.

Changes in topological properties, noted through graph theoretical analysis, were exhibited at differing depths of consciousness. Global and local efficiencies are more suitable for weighted undirected graphs compared to path length and cluster index^45^. These indices are common properties for measuring information integration and segregation in the functional network^46^. Propofol disrupts the cortical integration both, within- and between-network connectivities^8,15^. Specifically, propofol induces a breakdown of information integration between the fronto-parietal regions^16^. We observed that the global and local efficiencies increased for the delta band during transition into unconsciousness. Some studies show that the local efficiency for the delta band is increased in disorders of consciousness compared to that in healthy controls^47^. In other words, the changes of functional networks during unconsciousness are more globally integrated and less locally segregated for the delta band.

The alpha activity corresponded with the levels of consciousness^7^. With respect to functional network, the alpha connectivity is weaker during unconsciousness^21,22,48^. The global efficiency also decreases during transition into unconsciousness^20^. However, our study did not identify that alpha connectivity differentiates the conscious states. One possibility is that the concentration of propofol may not be high enough to result in changes of alpha connectivity. Moreover, when we performed comparisons of wakefulness and unconsciousness with the exception of transitions, similar to other studies, a significant difference between consciousness and unconsciousness was investigated in our results. Specifically, in the alpha band, parietal wPLI was significantly reduced during propofol-induced unresponsiveness compared to baseline (*p* = 0.035). There were no significant differences in frontal and fronto-parietal wPLI. This result is consistent with our argument that changes of brain dynamics with the levels of consciousness are directly related to the parietal and not the frontal region. It is also similar to recent studies indicating decrease in alpha connectivity during unconsciousness^20,21^.

Our study has some limitations. Strictly speaking, our study is not about the alternation of conscious states, but the transition to and from loss of responsiveness. Although the most frequently used experimental definition of anaesthesia-induced consciousness is in-line with the loss of behaviour^7,20^, unresponsiveness is not equivalent to unconsciousness. Even after losing responsiveness, the brain activity and connectivity continue to change with higher propofol concentrations^7,29,49^ potentially further limiting consciousness. In this study, we did not ask whether the subjects had conscious experiences during propofol rather focusing on the behavioural responsiveness as a surrogate for consciousness in real time. To this end, the subjects did not describe experiences on emergence from propofol. Future studies should use structured approaches to assess this.

We used the popular experimental paradigm using the auditory stimuli to investigate the critical transition point between consciousness and unconsciousness^7^. Therefore, the possibility of auditory evoked potentials could not be excluded. However, the tones were presented infrequently with only time-amplitude tones per episode of unresponsiveness and hence they are unlikely to bias the responses significantly.

Also, we did not explicitly make use of repeated transitions within individuals. Nonetheless no significant temporal and spatial changes in the same states across repeated transitions were obtained using one-way ANOVA (Supplementary Tables S5 and S6). Future studies should consider that neural inertia impedes the conserved behavioural state barrier in brain transitions between consciousness and unconsciousness.

In conclusion, we reported the changes of power, wPLI, and network properties during propofol-induced sedation using high-density EEG. We applied graph theoretical analysis for quantifying the levels of consciousness. Delta connectivity, and the associated traveling delta wave, appears a promising a signature of propofol-induced sedation, whereas beta connectivity marks the transitions into and out of consciousness. Our findings have potential clinical implications for the detection of level of consciousness, diagnosis of disorders of consciousness and also the mechanisms of consciousness itself.

## Methods

### Subjects

Ten healthy subjects (1 woman, 23–34 years of age) participated in this study. All subjects were members of American Society of Anesthesiologists (ASA) physical status 1 or 2. All clinical investigations were conducted at the Seoul National University Dental Hospital. This study was approved by the Institutional Board Review (KCT0001618). All experimental protocols, including any relevant details, were approved by the local ethics committee at the Seoul National University Dental Hospital. All methods were performed in accordance with the relevant guidelines and regulations by including a statement to this effect. Informed consent was obtained from all subjects. Subjects who were under 20 years of age, or with cardiovascular or respiratory history, or history of problems with anaesthesia, history of drug use, and any neurologic or psychiatric disease were excluded from this study.

### Experimental protocol

The subjects were requested to press a button intended for self-administration in response to an auditory stimulus (‘Press the button’). These stimuli were presented randomly every 9–11 sec to avoid instinctual pressing of the button. The system was programmed to deliver into a vein 0.3 mg/kg, on pressing the button. The patient-controlled intravenous infusion of propofol was commenced via a Perfusor Space syringe pump system (B. Braun Medical Inc., Melsungen, Germany). Unlike a computer-controlled infusion, which enables a direct control of the concentration of the anaesthetic, a patient-controlled infusion method required a lock-out interval of the injection for safety, to prevent an overdose^50^. The frequency of injection was defined as maximum, every 1 min. In other words, the propofol was injected by pressing the button, however, was not injected in case the button was pressed before the defined 1 min interval. On increasing the concentration of propofol, subjects were led to sedation. According to ASA, there were four levels of sedation such as minimal sedation, moderate sedation, deep sedation, and general anaesthesia. When subjects reached deep sedation, they stopped pressing the button. As the concentration of propofol dropped, subjects then recovered consciousness. After the transition from deep sedation to moderate sedation, subjects pressed the button again in response to auditory stimuli. The system started to re-inject propofol (Fig. 1). Changing the levels of sedation was repeated three or four times in the subject. The experiment was performed with simultaneous real-time EEG monitoring. For safety, bispectral index (BIS^TM^, Covidien, Mansfield, MA), blood pressure, oxygen saturation, electrocardiogram, and breathing frequency were measured. The BIS is widely used for monitoring the depths of anaesthesia, but it is not appropriate to measure the levels of consciousness clearly^51^. All sedation procedures were also continuously supervised by certified anaesthesiologists.

### EEG data acquisition and pre-processing

EEG data were collected using the BrainAmp EEG amplifier (Brain Products GmbH, Munich, Germany) with 62 Ag/AgCl channels (Brain Products GmbH, Munich, Germany). EEG data were referenced to FCz electrode and sampled at 1 kHz with impedance reduced to below 10 kΩ.

Continuous signals were down-sampled to 200 Hz and filtered between 1–50 Hz. EEG data were re-referenced to an average reference. Data from 56 channels over the scalp surface were obtained. The time window was equal to 10 sec in the five states (baseline, Trans_UN_, unresponsiveness, Trans_RES_, and recovery). The signals during baseline and recovery, regardless of the anaesthetic concentration and repeated transitions, were measured for 5 min and segmented into non-overlapping 10 sec. The data in the other states were epoched by 10 sec based on each control point. Data containing noise and artifacts such as eye movement and muscular activity were rejected. EEG signals were divided into six bands including delta (1–4 Hz), theta (4–8 Hz), alpha (8–12 Hz), sigma (12–15 Hz), beta (15–20 Hz), and gamma (20–40 Hz) by band-pass filtering. we This processing was performed using MATLAB (R2014b, The MathWorks, Natick, MA) based on EEGLAB^52^.

### Spectral power analysis

The epoched signals were used for calculating the mean spectral perturbation across trials during the levels of responsiveness. The baseline-normalized power spectrum was computed in each channel using Fast Fourier transforms. This spectral measure represents the mean relative changes in the amplitude of EEG frequency spectrum from baseline and also indicates brain dynamics at each frequency, not the averaged event-related potentials of the corresponding response.

To determine the roles in specific regions across the five states, we investigated the power spectrum in the frontal and parietal regions during the alternation of consciousness. After obtaining the power in each channel, the power spectrum over the frontal and parietal regions was averaged. The frontal region was regarded as 13 channels (FP1–2, AF3–6, F1–4, F7–8, and Fz), whereas the parietal region was defined as 14 channels (P1–8, Pz, PO3–4, PO7–8, and POz).

### Connectivity estimators

To calculate functional connectivity, the cross-spectrum of every pair of the channels was used to compute the wPLI^53^. The wPLI is a robust measure against the volume conduction and uncorrelated noise sources. This connectivity measure represents a phase-difference weighting normalization for decreasing movement artifacts in EEG. Specifically, signals related to sources that are evenly driven and are rapid in time are eliminated by running in phase space and weighting the phase differences of ±90 degree to the maximum. Therefore, the wPLI can only detect the phase-lag interactions from a complex coupled brain system and devoid of the artefacts that are intrinsically combined with brain activity^54^. For two signals, the wPLI is defined as:

1 $${\rm{\Phi }}=\,\frac{| E\{{\mathfrak{J}}\{X\}\}| }{E\{| {\mathfrak{J}}\{X\}| \}}=\frac{| E\{| {\mathfrak{J}}\{X\}| sgn({\mathfrak{J}}\{X\})\}| }{E\{| {\mathfrak{J}}\{X\}| \}}$$

${\mathfrak{J}}\{{X}\}$ is an imaginary component of cross-spectrum $X$ between two signals. The cross-spectrum $X$ is determined to ${Z}_{i}{Z}_{j}^{* }$. ${Z}_{i}$ is calculated by the complex value of Fourier transform of the signal $i$, whereas ${Z}_{j}^{* }$ is the complex conjugate of ${Z}_{j}$ of signal $j$. If the instantaneous phase of two signals leads or lags at all times, the value of ${\rm{\Phi }}$ is 1. On the other hand, if two signals have random phases lead/lag relationship, and then ${\rm{\Phi }}$ is 0^20^. After obtaining the wPLI between two channels, the frontal, parietal, and fronto-parietal wPLI were averaged. The linear correlation between power spectra and wPLI was calculated to investigate the relationship between power and wPLI.

The 56 × 56 subject-wise, band-wise wPLI matrices were generated. It is important to determine the correct connection density because an increase in connection density improves the possibility of false negatives in the network. In other words, high connection density can serve the removal of small density functionally relevant links^55^. To calculate the optimal connection density, we measured the mean global and local efficiencies values from 100 random graphs with 56 nodes. This thresholded density was calculated as the maximum value of the difference between global and local efficiencies (density = 0.2932)^56^. This connection density improves the independence between the global and local efficiencies while measuring the network properties. Applying the optimal connection density, each wPLI matrix was thresholded by retaining only the strongest 451 links. The connection strength above the thresholded density was preserved, whereas the other connection and all connections on the main diagonal strength were set to zero.

### Graph-theoretical analysis

Based on weighted adjacency matrix, the functional network can be measured by various properties. These properties were computed using the Brain Connectivity Toolbox^46^ and EEGNET^57^. We calculated the global efficiency, local efficiency, and small-worldness. These indices are crucial for understanding the information sharing, integration, and segregation among the brain system^58^. Global efficiency represents the total capacity for parallel processing of information and integrated processing^59^. Local efficiency indicates that information is effectively shared within the local network based on effectually segregated information processing^60^. Small-worldness also reflects on sharing the information processing within functional components using dense local links and performing the parallel processing using sparse but effective long-range links^61^ (see the supplementary method for more details). To explore the relationship between global and local efficiencies within the brain, we calculated the correlation between these.

### Statistical analysis

We measured the brain dynamics on repeated transitions between consciousness and unconsciousness. These repeated transitions were averaged with the assumption that these were comparable states. However, because of neural inertia, which is resistance of the brain in transitions between consciousness and unconsciousness as it creates a barrier to awareness^62^, the brain dynamics may not be the same from the first transition to/from loss of responsiveness as the second. So, we investigated that the brain dynamics varied statistically with the number of repeated transitions. The one-way ANOVA was used whether pre-processed EEG signals change within the same five states across repeated transitions, either spatially (in each channel) or temporally (in time), respectively. To infer the differences in power and wPLI across conscious states over the frontal and parietal regions, we also performed the one-way ANOVA. When appropriate, post hoc paired t-tests were used with Holm-Bonferroni correction. The one-way ANOVA was used for global efficiency, local efficiency, and small-worldness for the five states. To demonstrate posthoc analysis probing of main effects, paired t-test with Holm-Bonferroni correction was used. Statistical significance was set at level ${\rm{\alpha }}$ = 0.05.

### Data availability

The datasets generated during and/or analysed during the current study are available from the corresponding author on reasonable request.

## Electronic supplementary material

Supplementary information
