# Supplementary material for: Network Properties in Transitions of Consciousness during Propofol-induced Sedation
Source: Sci Rep. 2017 Dec 1;7:16791. doi: 10.1038/s41598-017-15082-5 (PMC5711919; doi:10.1038/s41598-017-15082-5)
Supplement: Supplementary file 1 — Supplementary information [file 41598_2017_15082_MOESM1_ESM.pdf]

Supplementary information

Network Properties in Transitions of Consciousness during Propofol-induced Sedation

Minji Lee[1], Robert D. Sanders[2], Seul-Ki Yeom[1], Dong-Ok Won[1], Kwang-Suk Seo[3], Hyun Jeong Kim[3], Giulio Tononi[4], and Seong-Whan Lee[1*]

[1]Department of Brain and Cognitive Engineering, Korea University, Seoul, Korea.

[2]Department of Anesthesiology, University of Wisconsin, Madison, WI, USA.

[3]Department of Dental Anesthesiology, Seoul National University Dental Hospital, Seoul, Korea.

[4]Department of Psychiatry, University of Wisconsin, Madison, WI, USA.

*Correspondence to: Seong-Whan Lee

1) Department of Brain and Cognitive Engineering, Korea University, Seoul, Korea

2) E-mail: sw.lee@korea.ac.kr

Supplementary discussion

Individual results of wPLI during the levels of responsiveness

The individual changes of wPLI were investigated for all frequencies during the levels of responsiveness (Supplementary Fig. S2). We focused on the delta and beta bands. In the beta band, the individual change of wPLI was noticeably increased only at the transition point between consciousness and unconsciousness. This was remarkably consistent across subjects. Although the delta connectivity statistically increased in the fronto-parietal interaction and parietal region during unresponsiveness. Most subjects showed clear changes of wPLI across five states; one did not. Comparing the unconscious state, including the transition into unresponsiveness or responsiveness, with the conscious state (baseline and recovery), the delta wPLI certainly increased on average during unconsciousness (Supplementary Fig. S1). Nonetheless, there was the outlier marked with a red plus sign on the changes of parietal wPLI in the Supplementary Figure S1c. The Sub03 did not respond purposefully to auditory stimuli during propofol-induced unresponsiveness but tried to wake up or moved severely as if there was sleepwalking. In Sub03, although the beta wPLI during the transition points into unresponsiveness or responsiveness rapidly increased, the delta wPLI at baseline continued to decrease until propofol infusion (propofol-induced unresponsiveness) and recovery of responsiveness. This feature could be thought that Sub03 was caused by not reaching the stable deep sedation state that does loss of spontaneous movement. Future experiments with a large number of subjects are expected to have smaller SD and even more clear changes of delta wPLI during the five states of propofol-induced unresponsiveness.

Supplementary methods

Traveling wave analysis

The negative peak is the criteria point to calculate the brain dynamics of the delta wave (slow oscillations) because this measure is pointed and simply distinguishable, and its timing is independent on the EEG baseline[1]. Specifically, the delta wave was detected on the midline channels placed along the anterior-posterior axis. The timing of negative peak was calculated at each electrode. In the case of the responsive state being the traveling wave, we also measured the velocity of the wave using correlation and linear regression. The distance was calculated on the anterior-posterior axis based on the Fpz electrode.

Graph theory

We calculated network properties based on the weighted phase lag index. Global efficiency, local efficiency, and small-worldness are appropriate for investigating integrated information, local segregation, and information sharing in the functional network.

Network integration indicates the availability to combine information of various brain areas and transmit information in the network[2]. Global efficiency is defined as the average inverse shortest (weighted) path length from one node to all other nodes in the functional network of global information integration.

$$E_{glob} = \frac{1}{N(N-1)} \sum_{i \neq j} \frac{1}{d_{ij}} \tag{1}$$

where N is the number of nodes composing the graph, and d_{ij} is the shortest path length between node i and node j. A higher value means shorter lengths and easily communicable in the network. Thus, an efficient global network means that the nodes are remarkably integrated and the path length between nodes is constantly shortened[3].

Local efficiency is the mean of all global efficiencies in every sub-graph.

$$E_{loc} = \frac{1}{N}\sum_i E_{glob}(A_i) \tag{2}$$

where A_i is the sub-graph of the first neighbours of node i. E_{loc} indicates the effectiveness of information delivery to the first neighbour of a certain node when this node is eliminated from the network. It can be interpreted as efficiency of information exchange in the local communities for measuring fault tolerance network between connected neighbours[4].

Altogether, small-worldness is defined as the ratio of clustering coefficient to characteristic path length.

$$\mathrm{S} = \frac{C/C_{rand}}{L/\,L_{rand}} \tag{3}$$

where C and C_{rand} indicate the clustering coefficients and L and L_{rand} indicate the characteristic path lengths in the tested network and a random network, respectively. A small-world network is characterised by high clustering coefficients and low characteristic path lengths. Therefore, this measure can reveal the balance between global integration and local segregation to examine self-organized critical dynamics in segregated and integrated processing within the brain[5].

Supplementary references

1 Massimini, M., Huber, R., Ferrarelli, F., Hill, S. & Tononi, G. The sleep slow oscillation as a traveling wave. *J. Neurosci.* **24**, 6862-6870 (2004).

2 Joudaki, A., Salehi, N., Jalili, M. & Knyazeva, M. G. EEG-based functional brain networks: does the network size matter? *PLoS One* **7,** e35673 (2012).

3 Stanley, M. L. *et al.* Changes in brain network efficiency and working memory performance in aging. *PLoS One* **10**, e0123950 (2015).

4 Alaerts, K. *et al.* Functional organization of the action observation network in autism: a graph theory approach. *PLoS One* **10**, e0137020 (2015).

5 Watts, D. J. & Strogatz, S. H. Collective dynamics of ‘small-world’networks. *Nature* **393**, 440-442 (1998).

Supplementary figures

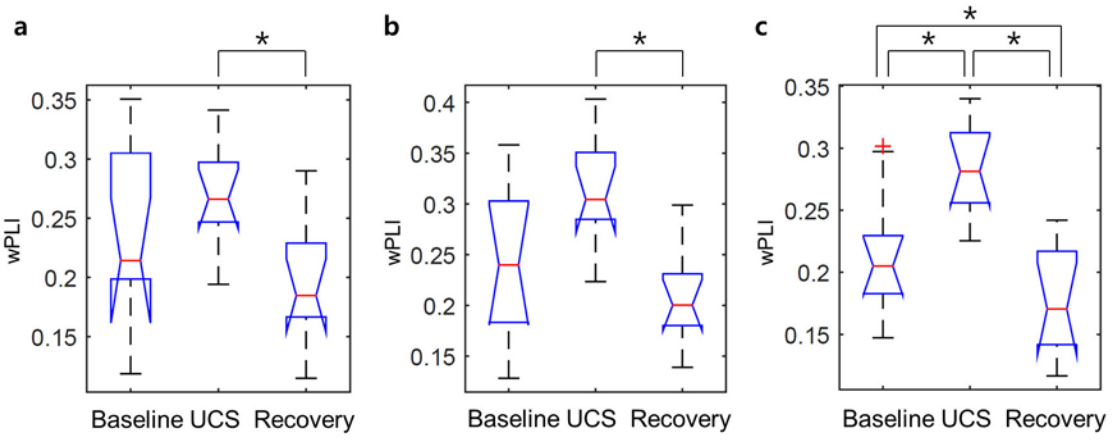

Supplementary Figure S1. The difference of delta wPLI between consciousness and unconsciousness. The wPLI in (a) frontal, (b) fronto-parietal, and (c) parietal regions was investigated. UCS indicates the average of transitions into responsiveness or unresponsiveness and propofol-induced unresponsiveness. The frontal region refers to 13 channels (FP1-2, AF3-6, F1-4, F7-8, and Fz), whereas the parietal region refers to 14 channels (P1-8, Pz, PO3-4, PO7-8, and POz). The asterisk means the significance with Holm-Bonferroni correction ($p < 0.05$).

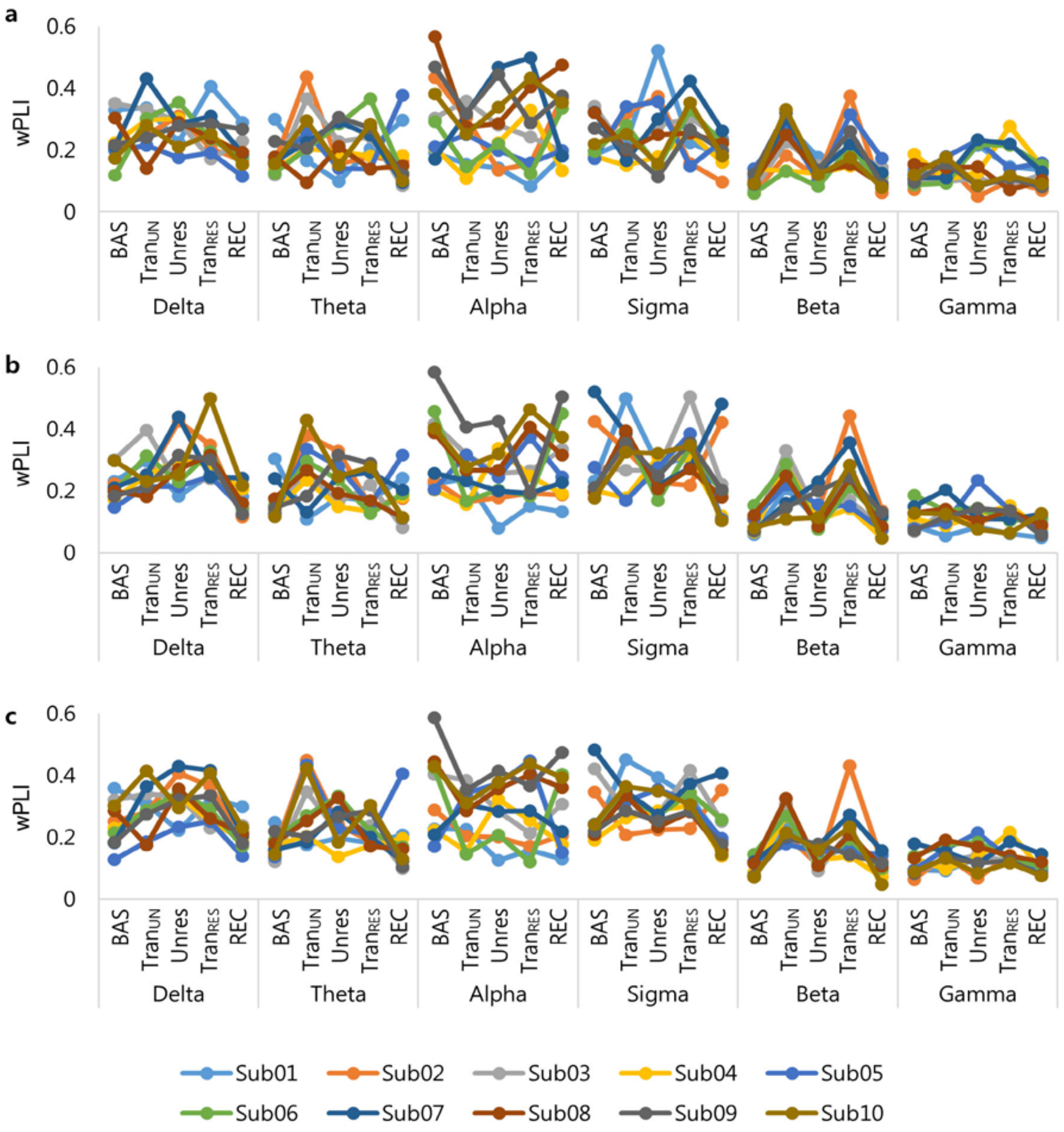

Supplementary Figure S2. The changes of the individual wPLI with the levels of responsiveness. It is plotted that (a) frontal wPLI, (b) fronto-parietal wPLI, and (c) parietal wPLI for all frequency bands. Each colour indicates the individual changes of wPLI. The frontal region refers to 13 channels (FP1-2, AF3-6, F1-4, F7-8, and Fz), whereas the parietal region refers to 14 channels (P1-8, Pz, PO3-4, PO7-8, and POz). BAS = baseline, $Tran_{UN}$ = transition into unresponsiveness, Unres = unresponsiveness, $Tran_{RES}$ = transition into responsiveness, REC = recovery.

Supplementary tables

Supplementary Table S1. Statistical *p*-value in spectra power at all frequencies. * represents the significance with Holm-Bonferroni correction ($p < 0.05$). $Trans_{UN}$ = transition into unresponsiveness, Unres = unresponsiveness, $Trans_{RES}$ = transition into responsiveness.

Region	Frequency	Baseline–$Trans_{UN}$	Baseline–Unres	Baseline–$Trans_{RES}$	Baseline–Recovery	$Trans_{UN}$–Unres	$Trans_{UN}$–$Trans_{RES}$	$Trans_{UN}$–Recovery	Unres–$Trans_{RES}$	Unres–Recovery	$Trans_{RES}$–Recovery
Frontal region	Delta	< 0.001*	< 0.001*	< 0.001*	0.135	0.002*	0.238	< 0.001*	0.001*	< 0.001*	< 0.001*
	Theta	< 0.001*	< 0.001*	< 0.001*	0.024	0.745	0.007*	< 0.001*	0.026	< 0.001*	< 0.001*
	Alpha	< 0.001*	< 0.001*	< 0.001*	0.219	0.026	0.638	< 0.001*	0.022	< 0.001*	< 0.001*
	Sigma	< 0.001*	< 0.001*	< 0.001*	0.271	0.074	0.324	< 0.001*	0.017	< 0.001*	< 0.001*
	Beta	< 0.001*	< 0.001*	< 0.001*	0.036	0.001*	0.008*	< 0.001*	0.689	< 0.001*	< 0.001*
	Gamma	< 0.001*	< 0.001*	< 0.001*	0.005*	0.065	0.632	< 0.001*	0.018	< 0.001*	< 0.001*
Parietal region	Delta	< 0.001*	< 0.001*	< 0.001*	0.376	0.009*	0.540	< 0.001*	0.004*	< 0.001*	< 0.001*
	Theta	< 0.001*	< 0.001*	< 0.001*	0.019	0.455	0.069	< 0.001*	0.065	< 0.001*	< 0.001*
	Alpha	0.001*	< 0.001*	< 0.001*	0.279	0.262	0.685	< 0.001*	0.688	< 0.001*	< 0.001*
	Sigma	< 0.001*	< 0.001*	< 0.001*	0.356	0.074	0.762	< 0.001*	0.045	< 0.001*	< 0.001*
	Beta	< 0.001*	< 0.001*	< 0.001*	0.028	0.013	0.296	< 0.001*	0.862	< 0.001*	< 0.001*
	Gamma	< 0.001*	< 0.001*	< 0.001*	0.121	0.104	0.445	< 0.001*	0.265	< 0.001*	< 0.001*

Supplementary Table S2. Statistical *p*-value in the delta wPLI between consciousness and unconsciousness. UCS indicates the average of transitions into responsiveness or unresponsiveness and propofol-induced unresponsiveness. The frontal region refers to 13 channels (FP1-2, AF3-6, F1-4, F7-8, and Fz), whereas the parietal region refers to 14 channels (P1-8, Pz, PO3-4, PO7-8, and POz). * represents the significance with Holm-Bonferroni correction ($p < 0.05$).

Region	Baseline - UCS		Baseline - Recovery		UCS - Recovery	
	t-value	*p-value*	*t-value*	*p-value*	*t-value*	*p-value*
Frontal region	-1.358	0.204	1.961	0.091	4.877	0.009*
Fronto-parietal interaction	-2.426	0.049	3.334	0.027	5.417	< 0.001*
Parietal Region	-4.782	0.011	2.751	0.029	6.652	< 0.001*

Supplementary Table S3. Statistical *p*-value in the wPLI during the levels of responsivenss. * represents the significance with Holm-Bonferroni correction ($p < 0.05$). $Trans_{UN}$ = transition into unresponsiveness, Unres = unresponsiveness, $Trans_{RES}$ = transition into responsiveness.

Region	Frequency	Baseline–$Trans_{UN}$	Baseline–Unres	Baseline–$Trans_{RES}$	Baseline–Recovery	$Trans_{UN}$–Unres	$Trans_{UN}$–$Trans_{RES}$	$Trans_{UN}$–Recovery	Unres–$Trans_{RES}$	Unres–Recovery	$Trans_{RES}$–Recovery
Frontal region	Beta	< 0.001*	0.042	0.001*	0.347	< 0.001*	0.523	< 0.001*	0.007*	0.211	0.002*
Fronto-parietal interaction	Delta	0.115	0.036	0.056	0.009	0.228	0.270	0.003*	0.577	0.002*	0.001*
	Beta	< 0.001*	0.019	0.003*	0.540	0.001*	0.487	< 0.001*	0.023	0.031	0.002*
	Gamma	0.039	0.121	0.007	0.082	0.762	0.873	0.002*	0.717	0.020	0.001*
Parietal Region	Delta	0.034	0.071	0.004*	0.022	0.523	0.226	0.002*	0.576	0.006*	0.001*
	Beta	< 0.001*	0.095	0.001*	0.954	0.024	0.542	0.001*	0.002*	0.017	< 0.001*

Supplementary Table S4. Statistical *p*-value of the network properties (global and local efficiencies) during the levels of consciousness.

* represents the significance with Holm-Bonferroni correction ($p < 0.05$). Trans_{UN} = transition into unresponsiveness, Unres = unresponsiveness, $\text{Trans}_{\text{RES}}$ = transition into responsiveness.

Properties	Frequency	Baseline–Trans_{UN}	Baseline–Unres	Baseline–$\text{Trans}_{\text{RES}}$	Baseline–Recovery	Trans_{UN}–Unres	Trans_{UN}–$\text{Trans}_{\text{RES}}$	Trans_{UN}–Recovery	Unres–$\text{Trans}_{\text{RES}}$	Unres–Recovery	$\text{Trans}_{\text{RES}}$–Recovery
Global efficiency	Delta	0.001*	0.006*	0.009*	< 0.001*	0.744	0.641	< 0.001*	0.420	< 0.001*	< 0.001*
	Beta	< 0.001*	0.005*	< 0.001*	0.349	< 0.001*	0.430	< 0.001*	< 0.001*	0.001*	< 0.001*
Local efficiency	Delta	0.003*	0.003*	0.024	< 0.001*	0.550	0.527	< 0.001*	0.214	< 0.001*	< 0.001*
	Beta	< 0.001*	0.001*	< 0.001*	0.406	< 0.001*	0.547	< 0.001*	0.001*	0.001*	< 0.001*

Supplementary Table S5. Statistical results of temporal changes in the same states across repeated transitions: There were no significant changes of EEG signals in time across the sedation cycle using one-way ANOVA ($p < 0.05$). The number of repeated transitions in Sub09 is one. Hence, statistical analysis was not performed for this subject. However, this does imply that the sedation cycle of Sub09 is one as we have defined that unconsciousness in the analysis should comprise of at least 3 min extending from LOR to ROR, in the manuscript. LOR = loss of responsiveness, ROR = recovery of responsiveness, $Trans_{UN}$ = transition into unresponsiveness, Unres = unresponsiveness, $Trans_{RES}$ = transition into responsiveness.

Subjects	Num. of repeated transitions	$Trans_{UN}$		Unres		$Trans_{RES}$	
		F-value	*p-value*	*F-value*	*p-value*	*F-value*	*p-value*
Sub01	4	0.018	0.997	0.272	0.846	0.023	0.995
Sub02	4	0.073	0.974	0.018	0.997	0.150	0.929
Sub03	3	0.100	0.905	0.004	0.996	0.056	0.946
Sub04	3	0.064	0.938	0.119	0.888	0.189	0.828
Sub05	2	0.016	0.898	0.001	0.971	0.040	0.841
Sub06	2	0.012	0.914	0.138	0.710	0.472	0.492
Sub07	2	0.014	0.906	0.301	0.583	0.218	0.641
Sub08	4	0.034	0.992	0.015	0.997	0.041	0.989
Sub09	1	–	–	–	–	–	–
Sub10	3	0.041	0.959	0.014	0.986	0.001	0.999

Supplementary Table S6. Statistical results of spatial changes in the same states across repeated transitions: There were no significant changes of EEG signals in each channel across the sedation cycle using one-way ANOVA (p < 0.05). The number of repeated transitions in Sub09 is one. Hence, statistical analysis was not performed for this subject. However, this does not imply that the sedation cycle of Sub09 is one; as we have defined that unconsciousness in the analysis should comprise of at least 3 min extending from LOR to ROR, in the manuscript. LOR = loss of responsiveness, ROR = recovery of responsiveness, $Trans_{UN}$ = transition into unresponsiveness, Unres = unresponsiveness, $Trans_{RES}$ = transition into responsiveness.

Subjects	Num. of repeated transitions	$Trans_{UN}$		Unres		$Trans_{RES}$	
		F-value	*p-value*	*F-value*	*p-value*	*F-value*	*p-value*
Sub01	4	0.102	0.959	0.903	0.441	0.202	0.895
Sub02	4	0.405	0.750	0.170	0.916	1.432	0.234
Sub03	3	0.219	0.804	0.046	0.955	0.128	0.880
Sub04	3	0.194	0.824	1.148	0.320	0.552	0.577
Sub05	2	0.477	0.491	0.009	0.924	0.917	0.340
Sub06	2	0.063	0.802	0.710	0.401	1.384	0.242
Sub07	2	0.242	0.624	0.731	0.394	1.769	0.186
Sub08	4	0.180	0.910	0.159	0.924	0.259	0.855
Sub09	1	–	–	–	–	–	–
Sub10	3	0.370	0.691	0.265	0.768	0.002	0.998
